# Acute Concurrent Exercise Improves Inhibitory Control Without Mediating the Role of Lactate: An Event-Related Potential Study

**DOI:** 10.1186/s40798-024-00809-2

**Published:** 2025-01-27

**Authors:** Ruei-Hong Li, Tai-Rui Chen, Nicholas D. Gilson, Marius Brazaitis, Yi-Ting Cheng, Hui-Fang Wu, Ji-Hang Lee, Yu-Kai Chang

**Affiliations:** 1 Department of Physical Education and Sport Sciences, National Taiwan Normal University, 162, Section 1, Heping E. Road, Taipei, 106 Taiwan; 2 School of Human Movement and Nutrition Sciences, The University of Queensland, St Lucia, QLD Australia; 3 Sports Science and Innovation Institute, Lithuanian Sports University, Kaunas, Lithuania; 4 Office of Physical Education, Soochow University, Taipei, Taiwan; 5 College of Sport Science, Sungkyunkwan University, Gyeonggi, South Korea; 6 Social Emotional Education and Development Center, National Taiwan Normal University, Taipei, Taiwan

**Keywords:** Concurrent training, Inhibition, Lactate, Mediation, P300

## Abstract

**Background:**

Concurrent exercise (CE), an emerging exercise modality characterized by sequential bouts of aerobic (AE) and resistance exercise (RE), has demonstrated acute benefits on executive functions (EFs) and neuroelectric P3 amplitude. However, the effect of acute CE on inhibitory control, a sub-component of EFs, and P3 amplitude remains inconclusive. Moreover, exploring the mechanisms underlying the effects of acute exercise on EFs contributes to scientific comprehension, with lactate recognized as a crucial candidate positively correlated with EFs. Therefore, this study aimed to determine the effects of acute CE on inhibitory control via behavioral and event-related potential approaches and to examine its potential mediational role on lactate.

**Methods:**

Seventy-eight adults (mean age = 22.95, *SD* = 1.75 years) were randomly assigned to either a CE, AE, or control (CON) group. Participants in the CE group engaged in 12-min of AE (40–59% of heart rate reserve [HRR]) coupled with 13-min of RE (1 set, with 75% of 10-repetition maximum, and 12 repetitions of 8 movements). The AE group participated in 25 min of AE (40–59% HRR). Prior to and following exercise onset, participants in both the CE and AE groups completed a 5-min warm-up and cool-down. Participants in the CON group read books for 35 min. Lactate concentrations were measured at timepoints of 0-, 17-, and 30-min relative to the treatment onset. Response time (RT) and accuracy in the Stroop test, as well as P3 amplitude, were assessed before and after the treatment.

**Results:**

The results revealed that both the CE and AE groups had significantly shorter RTs compared to the CON group, with no significant differences in accuracy among groups. A decrease in P3 amplitude was observed for the CE group compared to the AE and CON groups. The mediating effects of lactate between acute exercise and inhibitory control were insignificant.

**Conclusions:**

The findings suggest that both CE and AE improve inhibitory control and CE potentially enhances the efficient allocation of attention resources. The lack of a significant mediating effect of lactate warrants further investigation.

*Trial registration*: ClinicalTrials.gov, NCT06370286. Registered 12 April 2024—Retrospectively registered, https://clinicaltrials.gov/study/NCT06370286.

**Supplementary Information:**

The online version contains supplementary material available at 10.1186/s40798-024-00809-2.

## Key Points

- Acute concurrent exercise improves inhibitory control, and the effect is similar to aerobic exercise.
- Acute concurrent exercise potentially enhances the efficient allocation of attention resources.
- Lactate does not act as a mediator between acute exercise and inhibitory control.

**Supplementary Information:**

The online version contains supplementary material available at 10.1186/s40798-024-00809-2.

## Background

Younger adults, grappling with pivotal challenges as they assume new responsibilities and shape the trajectory of their adult lives, must rely on their capacity to adapt to dynamic environmental changes for success [1]. Executive functions (EFs) emerge as a critical ability for younger adults to navigate environmental changes, involving the control of thoughts and actions to achieve goals through the coordination of various cognitive functions. EFs encompass three core-domains, including *inhibitory control* (i.e., resist irrelevant information), *working memory* (i.e., maintain and manipulate information), and *cognitive flexibility* (i.e., shift between diverse rules or tasks) [2, 3]. Younger adults who exhibit superior EFs evidence lower risky behavior [4], reduced cardiovascular risk [5], higher conscientiousness [6], greater stress regulation [7], and better academic performance [8]. Exploring strategies that can facilitate EFs becomes crucial in fostering the resilience and adaptive capacities of younger adults to confront life challenges.

Acute bouts of exercise have been recognized as a feasible approach for improving EFs. This is supported by numerous meta-analyses [9–11] and strong evidence presented in the Physical Activity Guidelines for Americans (PAGA) [12]. While positive effects are recognized, there is a need to identify the optimal parameters of acute exercise [12] that can benefit EFs. To investigate this issue, Chang et al. [13] proposed the 3W1H framework of acute exercise and cognitive function which encompasses the *Who* (e.g., the characteristics of population), *What* (e.g., the intensity, duration, or modality of exercise; the domains of cognition), *When* (e.g., the time point of cognitive function assessment), and *How* (e.g., the mechanism between the acute exercise and cognition). Using this framework, researchers have begun to explore new modalities of exercise, such as concurrent exercise (CE), and investigate mechanisms that underpin the exercise – EF relationship [14].

CE characterized by back-to-back sessions of aerobic exercise (AE) and resistance exercise (RE) was initially employed as a strategic training approach for athletes facing the dual demands of cardiovascular endurance and muscular strength [15]. Recently, CE has emerged as a credible exercise modality, demonstrating promising health benefits such as weight loss [16], improved cardiorespiratory and muscular fitness [17], elevated brain-derived neurotrophic factor [18], reduced blood pressure [19], and a decreased inflammatory response [20]. These diverse physiological adaptations have also prompted researchers to investigate whether CE could also benefit EF.

The acute effects of CE on inhibitory control have involved varied designs and yielded ambiguous findings [21–23]. For example, Chang et al. [21] recruited 34 healthy young women, who participated in 30 min of CE comprising low-intensity RE (30% 1-repetition maximum [1-RM] with 12 repetitions) concomitant with moderate-intensity AE (50–60% heart rate reserve [HRR]). Similarly, Chen et al. [22] enlisted 15 healthy men to engage in CE involving moderate-intensity RE (4 sets, 4 upper body movements, 70% 10-repetition maximum [10-RM], and 10 repetitions) coupled with moderate-intensity AE (65% peak oxygen consumption [V̇O_2 peak_]) for 20 min. Both studies found a favorable impact on inhibitory control. However, Wen and Tsai [23] found no significant positive effect on inhibitory control in 32 obese women participating in 30 min of CE which alternated intervals of 1-min dumbbell RE with 1-min of moderate-intensity (55% HRR) AE (i.e., aerobic dance). Inconsistencies in results may stem from the sequential completing-session of CE, where completing one exercise before transitioning to another, rather than alternating between exercises, reveals advantages in EFs [14]. Given that interval aerobic and resistance exercises necessitate rapid movement transitions (e.g., recall movements), they impose a higher cognitive load, potentially exacerbating mental fatigue, which may diminish the cognitive benefits of acute exercise [24].

Exercise-induced lactate might be a possible physiological mechanism regarding the beneficial effects of acute exercise on inhibitory control. Lactate, a by-product of glycolysis, plays a critical role in activating the sympathetic nervous system and providing energy substrate for neurons [25], thereby influencing psychomotor arousal and cognitive processes [26–28]. Both CE and AE have been found to raise the concentrations of lactate [29], with CE showing a more marked rise than AE [30]. Exercise-induced lactate has been demonstrated to correlate positively with inhibitory control [31] as well as modify the delivery of neurotrophic factors [32, 33]. Recently, lactate has been suggested by Li et al. [14] to be a mediator in the connection between acute CE and cognitive flexibility. However, it is as yet unknown if lactate plays a mediating role with regard to how acute CE and AE affect inhibitory control.

Event-related potentials (ERPs), as indicators of cerebral potential alterations reflecting cognitive processes [34], have been extensively used to explore neuroelectric activation patterns in studies of acute exercise and EFs [35, 36]. The P3 component of ERP represents a endogenous stimulus-locked positive oscillation occurring around 300 to 700 ms after the stimulus onset and serves as an index of attention allocation during cognitive processes, particularly over parietal areas [37]. Unlike earlier ERP components (e.g., N1, P2) that primarily reflect sensory processing, or later components (e.g., N400, P600) associated with specific cognitive domains, P3 component offers a more general measure of cognitive resource allocation applicable across various EF tasks [37]. A recent meta-analysis observed that both AE and RE lead to elevated P3 amplitude following acute exercise [38]. Similarly, an augmentation in P3 amplitude subsequent to acute CE has been observed in both inhibitory control [23] and cognitive flexibility [14]. However, Wen and Tsai [23] utilized interval CE, while Li et al. [14] employed completing-session CE. The two studies also investigated different aspects of EFs. This raises the question of whether completing CE would result in a similar increase in P3 amplitude associated with inhibitory control, underscoring the necessity for further investigation.

Addressing a number of key research gaps, this study aimed to determine the acute effect of CE on inhibitory control and related P3 activation among younger adults. Additionally, the mediating effect of lactate in the relationship between acute exercise, encompassing both CE and AE, and inhibitory control was also examined. Our hypothesis posited that both CE and AE would lead to enhanced inhibitory control and P3 amplitude, wherein lactate would serve as a mediator in connecting acute exercise with inhibitory control.

## Methods

### Participants

Seventy-eight cognitively healthy participants (women *n* = 39; mean age = 22.72, *SD* = 1.62 years; men *n* = 39; mean age = 23.03, *SD* = 1.69 years) were recruited from universities in and around Taipei City, Taiwan. The sample size was determined by conducting a power analysis with G*Power 3.1 for a one-way, between-subjects ANCOVA design with a power of 0.8, *α* of 0.05, and effect size ($\upeta _{{\text{p}}}^{2}$) of 0.13 [14]. Participants met inclusion criteria as follows: aged between 20 and 30 years; able to exercise without undue risk (i.e., the first seven questions of the Physical Activity Readiness Questionnaire for Everyone [PAR-Q+] were answered “No”); right-handed dominance; typical or corrected-to-typical eyesight; absence of psychiatric or neurological ailments; limited physical activity in the previous month (i.e., < 150 min/week of moderate-intensity physical activity); non-obese status (body mass index [BMI] < 27 kg/m^2^); and no cardiorespiratory or neuromuscular conditions. Eligible participants were randomly assigned into one of three groups, including CE, AE, or the control group (CON) using a lottery draw. All participants provided informed consent and the study received ethical approval from the Research Ethics Committee of National Taiwan Normal University (Approval No. 202101HM005).

### Assessment

#### Cardiorespiratory Fitness

Cardiorespiratory fitness was assessed using the YMCA Submaximal Cycle Ergometer Test [39, 40]. This test, employing a submaximal approach and ergometry, offers significant safety advantages, and is particularly suitable for populations with irregular exercise habits. The detailed testing protocol comprised several stages: (a) Stage 1: Power was set at 25 watts (W) while maintaining a pedal cadence of 50 revolutions per minute (RPM) for 3 min, with the recording of heart rate (HR) in the final minute. (b) Stage 2: Adjustment of power was based on the HR recorded in the last minute of Stage 1. If HR exceeded 100, the power was set to 50 W; if HR was between 90 and 99, the power was set to 75 W; if HR was between 80 and 89, the power was set to 100 W; if HR was below 79, the power was set to 125 W. This stage was sustained for at least 3 min, with HR recorded every minute. If the HR difference between the last 2 min was greater than 5, indicating instability, an additional minute of testing was added until the HR difference was under 5, before progressing to the next stage. (c) Stages 3 and 4: Power increased by 25 W for the current stage, with advancement to the next stage contingent upon conditions similar to Stage 2. (d) Stage 5: The acquired HRs were utilized to estimate VO_2 peak_.

#### Muscular Fitness

Muscular fitness was evaluated using machine resistance fitness equipment, encompassing eight distinct movements: chest press, rowing, lat pull down, shoulder press, arm curl, leg extension, leg press, and leg curl. The comprehensive testing protocol encompassed the following stages: (a) Stage 1: Participants were directed to execute movements at the lowest load, completing 10–12 repetitions to familiarize themselves and warm up. (b) Stage 2: Participants engaged in sets with an approximate 20-pound load increment, aiming for 3–5 repetitions per movement. Rest intervals of around 1 min were adhered to between sets until the participant could not complete a single repetition. If a participant managed one repetition, that load was carried forward to Stage 3; otherwise, the load from the prior set was maintained. (c) Stage 3: Preceding this stage, participants rested for 3 min, utilizing the load determined in Stage 2. Participants exerted maximal effort until exhaustion, while the researcher recorded the number of repetitions and load. Subsequently, these figures were converted to 10-RM according to the prescribed training load table [41].

#### Stroop Test

Inhibitory control was assessed using a modified computerized version of the Stroop test [42], which was administered through Neuroscan Stim2 software (Compumedics Neuroscan, Charlotte, NC). The Stroop test was chosen for this study as it is a well-established paradigm for examining inhibitory control, particularly in relation to the P3 component of event-related potentials [23, 42, 43]. Additionally, using the Stroop test allows for direct comparison with previous research [23], enhancing the comparability and generalizability of our findings.

The test comprised four blocks, totaling 432 trials, with each block consisting of 108 trials. Within each block, three types of trials were presented in equal proportions: 36 neutral trials, 36 congruent trials, and 36 incongruent trials. The neutral trial involved displaying a square printed in red, green, or blue color. The congruent trial presented Chinese language prints in corresponding colors and words [i.e., 紅 (RED), 綠 (GREEN), or 藍 (BLUE)]. In contrast, the incongruent trial displayed Chinese language prints in different colors and words [e.g., 紅 (RED) printed in blue color]. Each block included 36 neutral trials, 36 congruent trials, and 36 incongruent trials. Participants were instructed to respond promptly and accurately to the color of the stimulus presented. Subsequently, the mean response time (RT) of correct trials and accuracy for each Stroop condition were examined. Moreover, we also calculated the Stroop effect (i.e., incongruent–congruent).

### Heart Rate and Rating of Perceived Exertion

A HR monitor (H10; Polar, Finland) was used to assess HRs, while Borg’s rating of perceived exertion (RPE) scale was applied to determine subjective exertion levels (scale of 6–20) [44]. Four HR metrics were derived, including baseline HR, pre-treatment HR, treatment HR, and post-treatment HR.

Baseline HR was recorded during a 5-min period while the participant remained at rest. Pre-treatment HR was recorded before and after the participant performed the pretest of the Stroop test. For the CE group, treatment HR was recorded at 2-min intervals during AE, and both before and after each movement of RE. In the AE group, treatment HR was recorded at 2-min intervals during AE. For the CON group, treatment HR was recorded before and after the book-reading activity. Post-treatment HR was recorded before and after the participant performed the Stroop test. The application of the RPE scale coincided with the intervals at which the treatment HR measurements were conducted.

### Electroencephalography Recordings and Analyses

A 32-channel electrode cap (Quik-Cap Neo Net; Compumedics Neuroscan, USA) was used to acquire neuroelectric activity from the scalp. Data collection and real-time processing were conducted using CURRY 8 Data Acquisition and Online Processing software (Compumedics Neuroscan, USA) and the Neuroscan SynAmps2 amplifier (Compumedics Neuroscan, USA). Electrode positions of the cap adhered to the International 10–10 System. Real-time neuroelectric activity was referenced to an electrode (i.e., Ref) between Cz and CPz electrodes and grounded to the AFz electrode. Electrooculography (EOG) activity was tracked using extra electrodes positioned vertically above and below the left eye orbit (VEOG; bipolar vertical EOG) and horizontally at the outer canthus of each eye (HEOG; bipolar horizontal EOG). Throughout the recording, the impedance of each electrode was kept below 10 kΩ. Real-time neuroelectric activity was digitized at a sampling rate of 1000 Hz, subjected to amplification at 500 times, and underwent a 60-Hz notch filter.

The offline neuroelectric activity analyses adhered to ERP guidelines [45] and were conducted using Matlab (2023, Mathworks Inc.), EEGLAB toolbox (version 2023.0) [46], and ERPLAB toolbox (version 10.0) [47]. Initially, the offline neuroelectric activity was downsampled to 250 Hz, bandpass filtered (i.e., 0.1–30 Hz half-amplitude cut-off, 12 dB/oct roll-off), and notch filtered at 60 Hz using the Cleanline plugin [48]. Artifact Subspace Reconstruction method was then applied to correct the data [49] and the data were saved as the “pre-ICA” dataset. To obtain clear eyeblinks signals through Independent Component Analysis (ICA), data must undergo noise reduction processing. The data were downsampled to 100 Hz and were filtered by a frequency band (i.e., 1–30 Hz half-amplitude cut-off, 48 dB/oct roll-off). Artifacts were automatically rejected (i.e., 500 µV threshold with 1000 ms time windows), and VEOG, HEOG, periocular, and the bad channels were removed. Subsequently, ICA was executed. The calculated ICA weights were integrated into the “pre-ICA” dataset, and saved as the “post-ICA” dataset for further processing. Blink artifacts of the “post-ICA” dataset were corrected using the “icablinkmetrics” plugin [50], and then were re-referenced using mastoid channels (M1, M2) and bad channels were spherically interpolated. Subsequently, the data were epoched (− 200 to 800 ms), baseline-corrected (− 200 to 0 ms), and epochs exceeding ± 150 µV and ± 100 µV peak-to-peak amplitude with 100 ms moving window were removed. Finally, the P3 amplitude was computed as the mean amplitude at the Pz channel between 300 and 600 ms post-stimulus for subsequent analyses [51].

### Treatment

#### Concurrent Exercise

The participants commenced the session by undergoing a 5-min warm-up on a cycle ergometer at 70 RPM. The initial load was set at 25 W/min and progressively incremented until reaching the target HR range of 40–59% of HRR during the last minute of the warm-up. Subsequently, participants engaged in AE on the cycle ergometer, maintaining a target HR for 12 min at 70 RPM. Following the AE, participants performed RE involving eight movements: chest press, rowing, lat pulldown, shoulder press, arm curl, leg extension, leg press, and leg curl. Each movement consisted of 12 repetitions executed at moderate intensity, specifically set at 70% of their 10-RM. The duration of the RE regimen spanned approximately 13 min, incorporating a 1-min rest interval between each movement. Participants concluded the session with a 5-min cool-down phase and full-body stretching exercises. The CE treatment and intensity were designed based on previous studies that demonstrated cognitive benefits [14].

#### Aerobic Exercise and Control

In the AE group, the participants underwent a 5-min warm-up identical to that of the CE group. Following this, they engaged in AE for 25 min, maintaining the target HR at 70 RPM on a cycle ergometer. To conclude, participants performed a 5-min cool-down identical to that of the CE group. The AE treatment and intensity were designed based on previous studies that demonstrated cognitive benefits [14]. In the CON group, participants remained comfortably seated and devoted 35 min to reading a book associated with exercise and health.

### Procedure

**Fig. 1:**
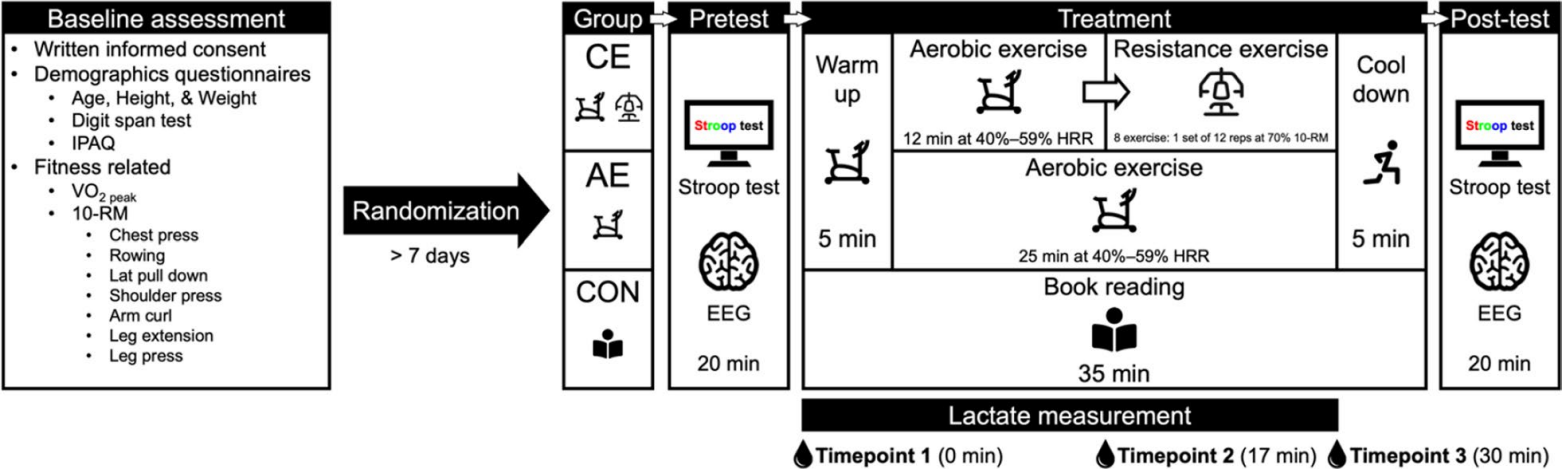
An overview of the experimental framework and investigative procedure. IPAQ, International physical activity questionnaire; 10-RM, 10-repetition maximum; CE, concurrent exercise group; AE, aerobic exercise group; CON, control group; EEG, electroencephalography; rep, repetition; HRR, heart rate reserve

Participants were scheduled for two laboratory visits, with a minimum gap of 7 days between each visit (refer to Fig. 1). Prior to each visit, participants were advised to avoid alcohol and caffeine for at least 6 h and to abstain from engaging in exercise for 12 h. In the initial visit, participants completed the written informed consent, PAR-Q + [39], the Digit Span Forward and Backward test to assess short-term and working memory [52], and the International Physical Activity Questionnaire to record exercise volume (IPAQ) [53]. Participants also completed assessments for height, weight, as well as cardiorespiratory and muscular fitness.

For the second visit, participants adhered to a multi-step procedure. Initially, participants performed the Stroop test as a pretest while their electroencephalography (EEG) activity was recorded. Subsequently, participants underwent the designated treatment based on their assigned group. Finally, participants concluded the session by performing the Stroop test as a post-test, during which EEG activity was recorded once more. Lactate levels were assessed using fingertip samples measured with a lactate analyzer (The EDGE, Taipei, Taiwan) at three distinct time points: Timepoint 1 (prior to the commencement of treatment), Timepoint 2 (17 min after the initiation of treatment), and Timepoint 3 (30 min after the start of treatment).

### Statistical Analyses

A one-way ANOVA was employed to examine demographics among the groups (Group: CE vs. AE vs. CON), with means (*M*) and standard deviations (*SD*) presented in the results (SPSS version 29.0, IBM Corp., Armonk, NYA). To assess the acute exercise effect on inhibitory control (i.e., RT and accuracy) and P3 amplitude, one-way ANCOVAs were executed for post-tests of each Stroop condition treating the pre-test as a covariate. A mixed-model ANOVA with a 3 (Group: CE vs. AE vs. CON) × 3 (Time: Timepoint 1 vs. Timepoint 2 vs. Timepoint 3) was analyzed for lactate. For exercise manipulation, a mixed-model ANOVA with a 3 (Group: CE vs. AE vs. CON) × 4 (Time: resting HR, pre-treatment HR, treatment HR vs. post-treatment HR) design was analyzed for HR. When assumptions of sphericity were violated, Greenhouse–Geisser corrections were applied.

To explore the mediational role of lactate, the mediation analysis of the simple mediation model was applied by PROCESS Macro SPSS plugin [54]. The independent variable was encoded into a dummy variable, using CON group as a reference [55]. The mediator, lactate, was quantified using the incremental area under the curve (iAUC). This was calculated by subtracting the lactate concentration at Timepoint 1 from the concentrations at Timepoint 2 and Timepoint 3, disregarding values below the Timepoint 1 baseline, and applying the trapezoidal rule [56]. Dependent variables were the post-test of the Stroop test, with the pre-test as a covariate. Regression analyses from group to lactate (Path *a*: CE vs. CON, AE vs. CON), from lactate to inhibitory control (Path *b*), and from group to inhibitory control (i.e., total effects [Path *c*]: CE vs. CON, AE vs. CON) were examined. If both paths a and b were significant [57], indirect effects (*a* × *b*: CE vs. CON, AE vs. CON) and direct effects (Path *c*: CE vs. CON, AE vs. CON) would be analyzed subsequently via the bootstrapping method for 5,000 times [58] and a 95% confidence interval (95% CI) was revealed. The mediating role of blood lactate was considered established if the 95% CI of the indirect effect did not span zero.

## Results

### Participant Demographics

Seventy-eight younger adults participated in the study (mean age = 22.95, *SD* = 1.75 years;* n* = 26 in each group). No significant differences in demographics were observed among the groups (*p*s > 0.05; i.e., IPAQ, estimated VO_2 peak_, and 10-RM). Detailed participant demographics are presented in Tables 1 and S1.

**Table 1:** Participant demographics among groups

	CE	AE	CON
*n*	26	26	26
Male/Female	13/13	13/13	13/13
Age (year)	22.73 ± 1.87	23.04 ± 1.48	22.85 ± 1.64
Height (m)	1.65 ± 0.08	1.67 ± 0.09	1.68 ± 0.07
Weight (kg)	60.83 ± 8.66	57.99 ± 7.44	61.74 ± 8.47
*Digit span*			
Forward (short-term memory)	14.81 ± 1.30	15.00 ± 1.20	14.65 ± 1.06
Backward (working memory)	10.96 ± 2.14	10.62 ± 2.76	10.15 ± 2.78
IPAQ (MET·min^−1^·weeks^−1^)	997.06 ± 857.92	606.99 ± 469.30	589.45 ± 385.56
Estimated VO_2 peak_ (ml·kg^−1^·min^−1^)	35.66 ± 5.97	34.06 ± 4.71	33.43 ± 3.40
*10-RM (lb)*			
Chest press	63.84 ± 36.77	57.37 ± 32.10	56.03 ± 31.68
Rowing	87.91 ± 23.73	88.78 ± 29.74	89.92 ± 28.48
Lat pull down	68.88 ± 24.30	64.79 ± 20.05	64.81 ± 21.62
Shoulder press	63.80 ± 20.29	58.15 ± 19.76	59.35 ± 21.51
Arm curl	43.50 ± 17.06	39.20 ± 16.91	39.59 ± 15.96
Leg extension	104.41 ± 33.30	103.64 ± 35.59	105.73 ± 26.36
Leg press	205.90 ± 54.19	198.23 ± 57.02	203.42 ± 51.41
Leg curl	67.97 ± 17.21	70.16 ± 23.04	68.44 ± 17.56

All values are *M* ± *SD*, excluding *n* and sex. CE, Concurrent exercise group; AE, aerobic exercise group; CON, control group; IPAQ, International Physical Activity Questionnaire; MET, metabolic equivalent of task; 10-RM, 10-repetition maximum; VO_2 peak_, peak oxygen consumption

### Stroop Test

#### Response Time

**Fig. 2:**
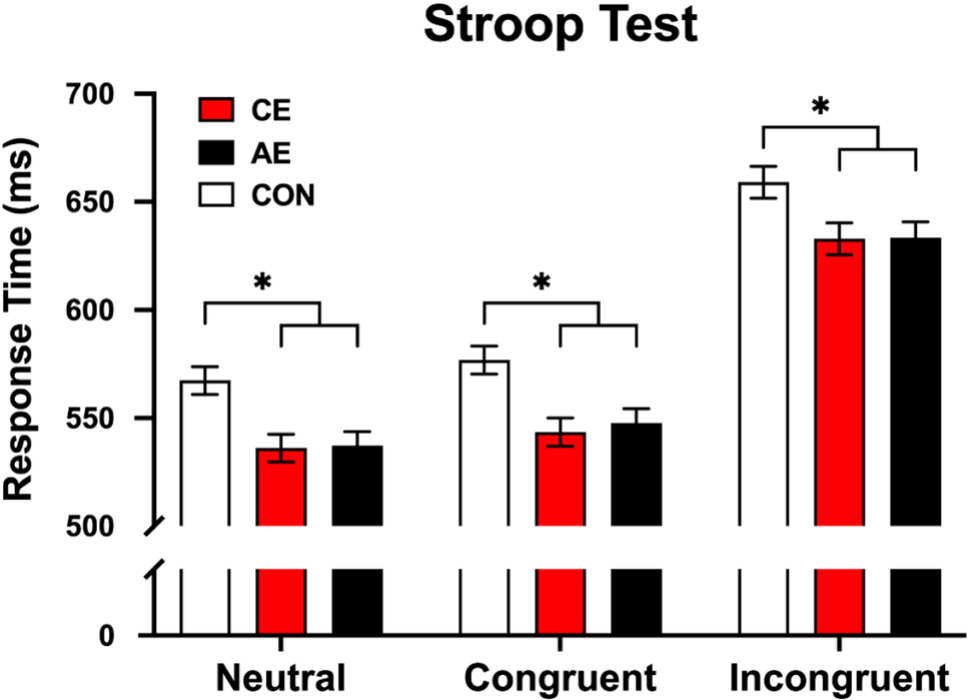
Acute exercise effect on inhibitory control regarding response time of the neutral, congruent, and incongruent conditions. Bar charts represent the mean and standard error of response time. CE, concurrent exercise group; AE, aerobic exercise group; CON, control group; *, Significant difference (*p* < 0.05)

A significant main effect was observed in the neutral, congruent, and incongruent conditions, respectively (*p*s < 0.001; Tables S2 and S3). Multiple comparisons revealed that both CE and AE groups had shorter RT compared to the CON group in neutral (CE: *p* = 0.003; AE: *p* = 0.004), congruent (CE: *p* = 0.002; AE: *p* = 0.007), and incongruent (CE: *p* = 0.043; AE: *p* = 0.048) conditions, with no significant difference between the CE and AE groups in each Stroop condition (*p*s = 1.000). A non-significant main effect was observed in Stroop effect (*p* = 0.179). Mean and standard error (*SE*) of RTs are shown in Fig. 2.

#### Accuracy

A non-significant main effect was observed in the neutral (*p* = 0.119), congruent (*p* = 0.082), and incongruent condition (*p* = 0.087); and Stroop effect (*p* = 0.107) (Tables S2 and S3).

#### P3 Amplitude

**Fig. 3:**
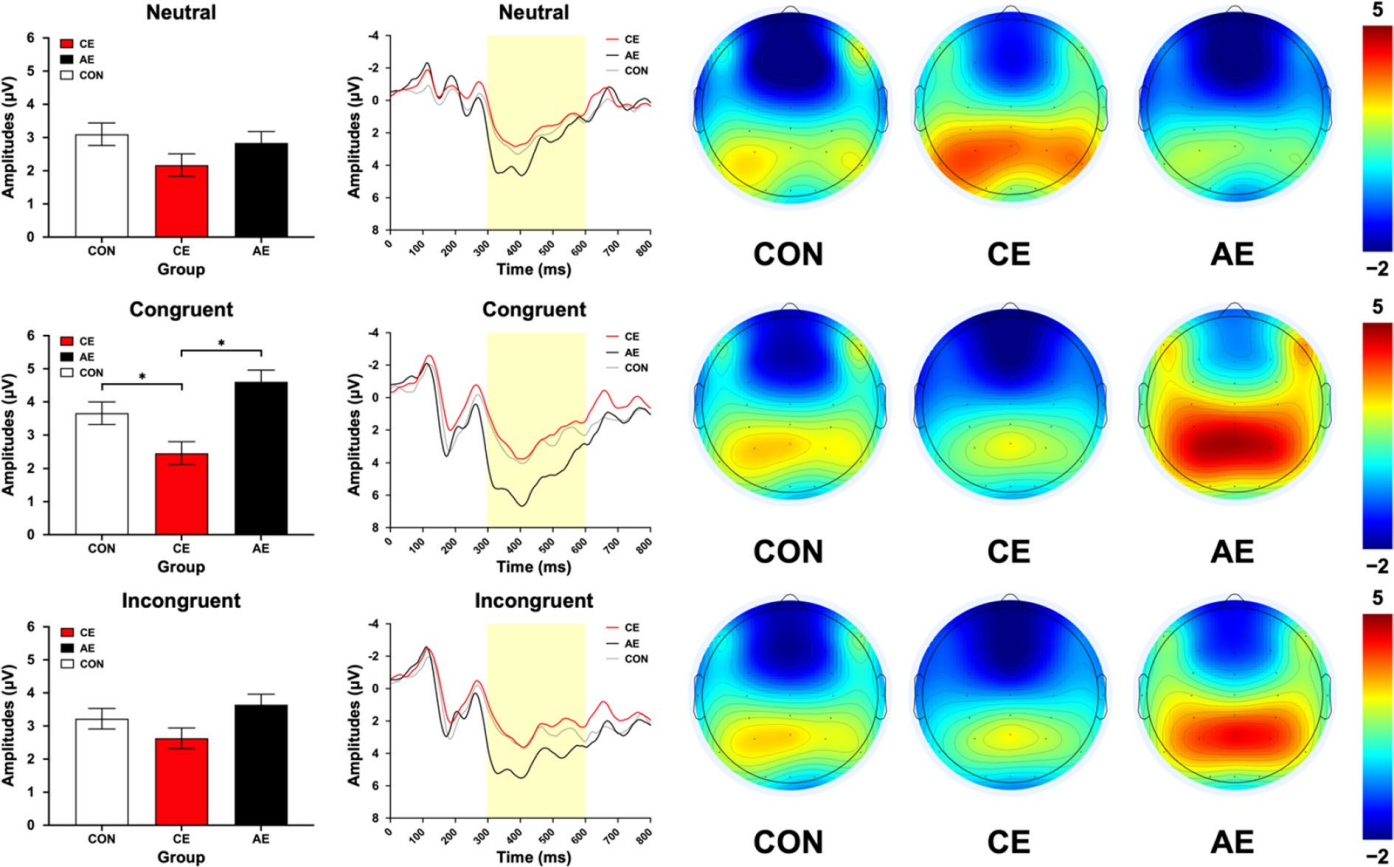
Acute exercise effect on inhibitory control regarding P3 amplitude of the neutral, congruent, and incongruent conditions. Bar charts represent the mean and standard error of P3 amplitude. Waveform charts represent P3 amplitude of post-test of the Stroop test between 0 and 800 ms. Brain scalp charts represent the P3 amplitude of post-test of the Stroop test between 0 and 800 ms among electrodes. CE, Concurrent exercise group; AE, aerobic exercise group; CON, control group; *, Significant difference (*p* < 0.05)

One-way ANCOVAs indicated no significant main effects of P3 amplitude among groups in the neutral (*p* = 0.135) and incongruent (*p* = 0.094) conditions; and Stroop effect (*p* = 0.052). However, a significant main effect was observed in the congruent condition (*p* < 0.001). Further multiple comparisons revealed that the CE group exhibited smaller P3 amplitude compared to both the AE (*p* < 0.001) and CON (*p* = 0.040) groups (Fig. 3, Tables S2, and S3).

### Lactate

A significant interaction of Group × Time as well as a main effect of Group and Time were observed (*p*s < 0.001; Tables S2 and S3). Multiple comparisons indicated that lactate was not significantly different among the groups at Timepoint 1 (*p*s > 0.05). However, both CE (*p* < 0.001) and AE (*p* < 0.001) groups revealed significantly higher lactate level compared to the CON group at Timepoint 2. The CE group had the significantly highest lactate level, followed by AE (*p* < 0.001) and CON (*p* < 0.001) groups at Timepoint 3. Within the CE group, lactate was significantly highest at Timepoint 3, followed by Timepoint 2, and Timepoint 1 (*p*s < 0.001). Within the AE group, lactate was significantly higher at both Timepoints 2 and 3 compared to Timepoint 1(*p*s < 0.001). No significant differences were observed among timepoints (*p*s > 0.05) within the CON group.

### Mediation of Lactate

#### Response Time

In the CE and AE groups, both path *a* and the total effect revealed significant regression in the neutral, congruent, and incongruent conditions (*p*s < 0.05), but path *b* was not significant (*p*s > 0.05, Table 2). Regarding the Stroop effect, only path *a* revealed significant regression (*p*s < 0.05).

**Table 2:** The mediation analyses of lactate for acute exercise effect on inhibitory control

	Group	Path *b*			Path *a*			Total effect		
		C	LCI	UCI	C	LCI	UCI	C	LCI	UCI
Response time										
Neutral	CE	− 0.03	− 0.23	0.16	81.22*	59.73	102.71	− 31.25*	− 49.40	− 13.10
	AE				58.48*	37.02	79.93	− 30.05*	− 48.18	− 11.93
Congruent	CE	0.00	− 0.20	0.20	81.46*	59.97	102.96	− 33.37*	− 51.75	− 14.98
	AE				58.52*	37.02	80.01	− 29.10*	− 47.49	− 10.71
Incongruent	CE	− 0.04	− 0.26	0.19	81.55*	60.05	103.05	− 26.15*	− 46.92	− 5.38
	AE				58.34*	36.81	79.88	− 25.71*	− 46.51	− 4.90
Stroop effect	CE	− 0.01	− 0.17	0.14	70.45*	49.27	91.63	12.90	− 0.79	26.60
	AE				46.98*	25.79	68.17	6.51	− 7.19	20.21
Accuracy										
Neutral	CE	0.00	− 0.01	0.01	87.17*	64.65	109.69	1.08	− 0.13	2.28
	AE				58.95*	37.74	80.15	0.66	− 0.47	1.80
Congruent	CE	0.00	− 0.01	0.01	81.43*	59.97	102.90	0.39	− 0.62	1.40
	AE				59.01*	37.43	80.59	1.13*	0.12	2.15
Incongruent	CE	0.02*	0.00	0.04	81.54*	60.04	103.04	2.00*	0.10	3.90
	AE				58.46*	36.90	80.02	1.96*	0.06	3.87
Stroop effect	CE	0.00	− 0.59	0.02	70.69*	49.18	92.21	2.03*	0.11	3.95
	AE				47.42*	25.85	68.98	0.63	− 1.30	2.55

CE, Concurrent exercise group; AE, aerobic exercise group; C, coefficients; LCI, lower limit of confidence interval; UCI, upper limit of confidence interval; *, significant effect (i.e., confidence interval does not include zero)

#### Accuracy

In the CE and AE groups, both path *a* and the total effect revealed significant regression in the neutral and congruent conditions (*p*s < 0.05, Table 2), but path *b* was not significant (*p*s > 0.05). Regarding the incongruent condition, path *a*, path *b,* and total effect revealed a significant regression, but the indirect effect was not significant in both the CE (95% CI: − 0.09–2.99) and AE (95% CI: − 0.06 to 2.40) groups (Table 2). Regarding the Stroop effect, only path *a* revealed significant regression of both CE and AE (*p*s < 0.05), and there was a significant regression of the total effect of CE (*p* < 0.05).

### Heart Rate and RPE

A significant interaction of Group × Time as well as a main effect of Group and Time were observed for HR (*p*s < 0.001; refer to Tables S2 and S3). Multiple comparisons indicated that resting and pre-treatment HRs were not significantly different among the groups (*p*s > 0.05). For treatment HR, the AE group had a significantly higher HR level compared to the CE group (*p* = 0.001), with the CE group significantly higher than the CON group (*p* < 0.001). For post-treatment HR, both CE (*p* < 0.001) and AE (*p* < 0.001) groups revealed significantly higher HR levels compared to the CON group. Within the CE group, treatment HR was significantly the highest, followed by post-treatment HR, pre-treatment HR, and resting HR (*p*s < 0.001). Similarly, within the AE group, treatment HR was significantly the highest, followed by post-treatment HR, pre-treatment HR, and resting HR (*p*s < 0.001). Within the CON group, resting HR was significantly the lowest compared to pre-treatment HR (*p* < 0.001), treatment HR (*p* < 0.001), and post-treatment HR (*p* = 0.001). No significant differences were observed among pre-treatment HR, treatment HR, and post-treatment HR (*p*s > 0.05).

A non-significant main effect of group was observed for RPE (*p* = 0.479; refer to Tables S2 and S3), indicating that there was no difference in RPE between CE and AE.

## Discussion

The present study examined the effects of acute CE and AE on inhibitory control, utilizing both behavioral and neuroelectric assessments among younger adults. We also investigated the mediating role of lactate in the relationship between acute exercise and inhibitory control. Our findings revealed that acute CE, combining both AE and RE, increased inhibitory control by reducing RT across all Stroop test conditions, regardless of accuracy. Beneficial effects for Stroop data were similar for acute AE. Differences in P3 amplitude were observed only in the congruent condition, where the amplitude of the CE group were significantly smaller than those of AE and CON groups. However, no mediating effects of lactate for acute exercise and inhibitory control were observed.

Relative to the hypothesis, our findings indicate that acute AE improved inhibitory control by reducing RT. These improvements were accompanied by no significant differences in accuracy, indicating that this enhancement is unlikely to be associated with a trade-off between speed and accuracy [59]. The results replicated previous studies demonstrating the positive effect of acute AE on inhibitory control among younger adults [60, 61]. A meta-analysis, synthesizing data from 55 effect sizes, also supports this, indicating a small and positive effect of acute moderate AE on inhibitory control (Hedges’ *g* =  − 0.26, 95% CI − 0.34 to − 0.18) [62]. The design of acute AE in the present study, structured as a 25-min moderate-intensity exercise, is noteworthy for its alignment with the PAGA recommendation regarding the facilitation of EF enhancements [12, 63]. Taken together, these findings suggest that acute AE, characterized by moderate intensity over a duration of 20 to 30 min, holds the potential to elicit a favorable effect on inhibitory control.

Similarly, our findings also indicate acute CE facilitated inhibitory control and this aligns with previous studies showing increased inhibitory control with acute CE [21, 22]. While Wen and Tsai [23] found no effect on inhibitory control after an interval-session of CE, the inconsistency may be attributed to the need for CE to incorporate both completing-sessions of AE and RE, if benefits on EFs are to be elicited [14]. This inconsistency might be explained by the fact that interval aerobic and resistance exercises necessitate rapid movement transitions (e.g., recall movements). Such exercises impose a higher cognitive load, potentially exacerbating mental fatigue, which may diminish the cognitive benefits of acute exercise [24]. Notably, Quintero et al. [64] tested CE that combined both AE with intensity interval form and RE, and reported enhanced inhibitory control. This finding not only supports that a complete CE is essential for enhancing EFs but also supports the view that intensity interval forms of single AE, when integrated into a comprehensive CE session, could also be effective. Therefore, our findings extend previous research, emphasizing that the design of CE should include a completing-session of both AE and RE for optimal EFs benefits.

Contrary to our hypothesis, there were no significant differences in P3 amplitude between the AE and control treatments. The inconsistency might be attributed to differences in cardiorespiratory fitness levels. For example, increased P3 amplitude following acute AE were observed among individuals with higher cardiorespiratory fitness levels (49.18 ± 7.57 ml·kg^−1^ min^−1^) [65], whereas our participants demonstrated lower cardiorespiratory fitness levels (34.38 ± 4.84 ml·kg^−1^ min^−1^). Moreover, Tsai et al. [66] compared acute AE effects on P3 amplitude among younger adults with varying levels of cardiorespiratory fitness and found that the high-fitness group displayed larger P3 amplitude compared to both the low-fitness and control groups, suggesting a potential association between cardiorespiratory fitness levels and differences in P3 amplitude. A meta-analysis by Kao et al. [38] supports this view and found the absence of a noticeable effect of acute exercise on P3 amplitude in individuals with low cardiorespiratory fitness.

It is noteworthy that while our focus was on cardiorespiratory fitness, other exercise-related factors may also contribute to the observed results. Kao et al. [38] reported that acute exercise with parameters similar to those employed in our study (i.e., AE, moderate intensity, younger adults, between-subjects design with pre- and post-tests, and randomization) typically elicits increases in P3 amplitude. However, this effect was not observed in individuals with lower cardiorespiratory fitness. Given the alignment between our exercise protocol and those associated with P3 amplitude enhancement in previous literature, we posit that cardiorespiratory fitness remains a primary factor in elucidating our findings.

One of our novel findings is the reduction in P3 amplitude observed following acute CE compared to acute AE and the control treatment, particularly noted in the congruent condition. Although this result was inconsistent with our hypothesis, it might indicate that CE could efficiently modulate attentional resources based on cognitive demands [67]. Li et al. [14] found that acute CE produced larger P3 amplitude compared to control treatment in high cognitive demands task (i.e., cognitive flexibility), suggesting that acute CE might require increased attentional resources to meet higher cognitive demand conditions [37]. Moreover, our findings showed no difference in P3 amplitude between acute CE and control treatment in the incongruent condition (i.e., inhibitory control), suggesting acute CE maintains similar attentional resource but has better performance in a condition which involves a lower cognitive domain than cognitive flexibility. In the congruent condition, which involves lower cognitive demands than in the incongruent condition, acute CE triggered smaller P3 amplitude compared to acute AE and the control treatment. These findings regarding P3 amplitude suggest a more efficient allocation of attentional resources following acute CE, possibly leading to enhanced inhibitory control.

The observed differences in P3 amplitude between CE and AE may be attributed to their distinct movement patterns and neural engagement. CE, combining AE and RE, likely activates a broader range of neural networks associated with EFs [68]. This comprehensive engagement could serve as an effective cognitive primer, leading to more efficient post-exercise neural processing. This efficiency manifested as improved inhibitory control with reduced neural activation (smaller P3 amplitude) following CE. AE alone may not provide the same range of motor engagement or priming effect, explaining the absence of similar efficiency gains. Thus, the unique combination of exercise modalities in CE appears to contribute to enhanced post-exercise cognitive efficiency, balancing the varied demands during exercise with improved performance afterward.

Taken together, our results suggest that following acute CE, attentional resources may be more efficiently managed, necessitating fewer resources particularly in conditions with lower cognitive demands. From a practical perspective, these data suggest that acute CE may help EF function in young adults. For instance, regular participation might mitigate interference during focused studying, and during demanding and critical work situations, CE might benefit transition and flexibly between different perspectives and ideas.

The findings of the present study indicated that lactate played no mediating role between acute exercise and inhibitory control. In contrast to our findings, Li et al. [14] showed that lactate mediated the impact of both acute AE and CE on EF, specifically cognitive flexibility. This discrepancy may be explained by the varying cognitive demands of different EF components. Cognitive flexibility necessitates concurrent engagement of both inhibitory control and working memory [67, 69], potentially imposing a greater cognitive demand compared to inhibitory control alone. Given that lactate serves as a brain energy source [28] and its demand increases proportionally with cognitive load [70], the absence of lactate mediation in our study may be attributed to the relatively lower cognitive demands of inhibitory control tasks, which might not necessitate substantial lactate utilization to support EF.

Moreover, the arousal hypothesis might be a possible mechanism to explain the positive effect of acute CE on inhibitory control [26]. Previous studies have found that both acute AE and RE could reduce the concentration of cortisol and improve inhibitory control [71], which is closely related to arousal levels. According to the inverted-U hypothesis, an optimal level of arousal may lead to optimal cognitive performance [26]. The dual stimulus of aerobic and resistance components in CE could lead to a similar cortisol response, potentially optimizing the relationship between arousal and inhibitory control. Therefore, the combination of AE and RE in CE may result in a favourable arousal state, contributing to the observed improvements in inhibitory control.

Our study has a number of strengths and is the first to examine the impact of acute CE on inhibitory control in younger adults, using a comprehensive set of measures that included both behavioral and neuroelectric assessments. We also explored the mediating influence of lactate on the relationship between acute exercise, inhibitory control, and P3 amplitude, and aimed to elucidate the relationships among these factors. The study design was strong and incorporated a randomized between-group approach, encompassing both sexes, and concurrently assessed cardiorespiratory and muscular fitness. This comprehensive approach allowed for the consideration of multiple factors, distinguishing our research from previous studies [21–23].

Several limitations should also be acknowledged. Firstly, this study focused solely on the impact of CE on inhibitory control in an “aerobic-resistance order.” The effects of acute CE on the “resistance-aerobic order” in inhibitory control remain unexplored. Past research has shown that different sequences of acute CE can elicit distinct responses in growth hormones [72], which are associated with inhibitory control [73]. Moreover, as the forms and doses of AE and RE might have different effects on EF [64, 74, 75] and related hormone response (e.g., cortisol [76]), future studies should investigate various exercise parameters in CE to determine their optimal combinations for enhancing EF. Secondly, this study inferred effects on inhibitory control, leaving the impact on working memory and planning, which are additional components, unexplored. Future studies should investigate the benefits of acute CE on working memory to explore both the similarities and differences across various core domains of EFs. This approach will provide a more comprehensive understanding of the positive effects of acute CE on EFs. Finally, it is important to note that, while we controlled for many factors, the ecological validity in practical settings still needs to be considered, such as the impact of exercise on work-related cognitive functions [77]. Transition from laboratory settings to real-world conditions is necessary.

## Conclusions

The study findings underscore the beneficial impact of acute CE on inhibitory control in younger adults, emphasizing its role in efficiently allocating attentional resources, although lactate does not appear to mediate these advantageous effects. This research provides valuable insight into the potential advantages of CE for younger adults aiming to improve inhibitory control and also highlights areas for further investigation into the underlying mechanisms that influence inhibitory control following acute CE.

## Supplementary Information

Additional file1.

## Data Availability

Data will be made available on reasonable request from the corresponding author.

## Abbreviations

1-RM: 1-Repetition maximum
10-RM: 10-Repetition maximum
AE: Aerobic exercise
BMI: Body mass index
CE: Concurrent exercise
CON: Control group
EEG: Electroencephalography
EFs: Executive functions
EOG: Electrooculography
ERPs: Event-related potentials
HR: Heart rate
HRR: Heart rate reserve
ICA: Independent Component Analysis
PAR-Q+: Physical activity readiness questionnaire for everyone
RE: Resistance exercise
RPM: Revolutions per minute
VO_2 peak_: Peak oxygen consumption
W: Watts
