# Supplementary material for: Acute Concurrent Exercise Improves Inhibitory Control Without Mediating the Role of Lactate: An Event-Related Potential Study
Source: Sports Med Open. 2025 Jan 27;11:12. doi: 10.1186/s40798-024-00809-2 (PMC11772916; doi:10.1186/s40798-024-00809-2)
Supplement: Supplementary file 1 — Additional file1. [file 40798_2024_809_MOESM1_ESM.docx]

**Supplementary Material**

**Table S1** One-way ANOVAs of demographics among groups

**Table S2** One-way ANCOVAs of behavioral and neuroelectric assessments of inhibitory control and two-way ANOVAs of heart rate and lactate among groups

**Table S3** Estimated values of behavioral and neuroelectric assessments of inhibitory control, and values of heart rate and lactate

**Table S1** One-way ANOVAs of demographics among groups

| Measures | *F* | *df* |  | *p* | $\text{η}_{\text{p}}^{\text{2}}$ |
| --- | --- | --- | --- | --- | --- |
| Age (years) | 0.23 | 2 | 75 | .799 | .006 |
| Height (m) | 0.79 | 2 | 75 | .456 | .021 |
| Weight (kg) | 1.48 | 2 | 75 | .235 | .038 |
| Digit Span Test |  |  |  |  |  |
| Forward | 0.92 | 2 | 75 | .404 | .024 |
| Backward | 0.64 | 2 | 75 | .529 | .017 |
| IPAQ (MET-min·week^−1^) | 2.72 | 2 | 75 | .072 | .068 |
| Estimated V̇O_2 peak_ (ml·kg^−1^·min^−1^) | 1.48 | 2 | 75 | .235 | .038 |
| 10-RM (lb) |  |  |  |  |  |
| Chest press | 0.40 | 2 | 75 | .671 | .011 |
| Rowing | 0.04 | 2 | 75 | .966 | .001 |
| Lat pull down | 0.27 | 2 | 75 | .766 | .007 |
| Shoulder press | 0.62 | 2 | 75 | .54 | .016 |
| Arm curl | 0.53 | 2 | 75 | .591 | .014 |
| Leg extension | 0.03 | 2 | 75 | .972 | .001 |
| Leg press | 0.14 | 2 | 75 | .874 | .004 |
| Leg curl | 0.09 | 2 | 75 | .913 | .002 |

IPAQ, International Physical Activity Questionnaire; MET, metabolic equivalent of task; 10-RM, 10-repetition maximum.

**Table S2** One-way ANCOVAs of behavioral and neuroelectric assessments of inhibitory control and two-way ANOVAs of heart rate and lactate among groups

| Measures | *F* | *df* |  | *p* | $\text{η}_{\text{p}}^{\text{2}}$ |
| --- | --- | --- | --- | --- | --- |
| Response time |  |  |  |  |  |
| Neutral | 7.57 | 2 | 74 | .001 | .170 |
| Congruent | 7.75 | 2 | 74 | < .001 | .173 |
| Incongruent | 4.12 | 2 | 74 | .020 | .100 |
| Stroop effect | 1.76 | 2 | 74 | .179 | .045 |
| Accuracy |  |  |  |  |  |
| Neutral | 2.20 | 2 | 74 | .119 | .056 |
| Congruent | 2.58 | 2 | 74 | .082 | .065 |
| Incongruent | 2.53 | 2 | 74 | .087 | .064 |
| Stroop effect | 2.31 | 2 | 74 | .107 | .059 |
| P3 amplitudes |  |  |  |  |  |
| Neutral | 2.06 | 2 | 74 | .135 | .053 |
| Congruent | 9.59 | 2 | 74 | < .001 | .206 |
| Incongruent | 2.45 | 2 | 74 | .094 | .062 |
| Stroop effect | 3.09 | 2 | 74 | .052 | .077 |
| Heart rate |  |  |  |  |  |
| Group × Timepoint | 127.23 | 5.303 | 198.845 | < .001 | .772 |
| Group | 61.52 | 2 | 75 | < .001 | .621 |
| Timepoint | 661.48 | 2.651 | 198.845 | < .001 | .898 |
| RPE |  |  |  |  |  |
| Group | 0.51 | 1 | 50 | .479 | .010 |
| Lactate |  |  |  |  |  |
| Group × Timepoint | 26.13 | 4 | 150 | < .001 | .411 |
| Group | 57.00 | 2 | 75 | < .001 | .603 |
| Timepoint | 73.34 | 2 | 150 | < .001 | .494 |

RPE, rating of perceived exertion.

**Table S3** Estimated values of behavioral and neuroelectric assessments of inhibitory control, and values of heart rate and lactate

|  | CE (*n* = 26) | | AE (*n* = 26) | | CON (*n* = 26) | |
| --- | --- | --- | --- | --- | --- | --- |
|  | *M* | *SE* | *M* | *SE* | *M* | *SE* |
| Response time (ms) |  |  |  |  |  |  |
| Neutral | 536.09 | 6.44 | 537.29 | 6.44 | 567.34 | 6.43 |
| Congruent | 543.47 | 6.53 | 547.74 | 6.53 | 576.84 | 6.52 |
| Incongruent | 632.95 | 7.37 | 633.39 | 7.38 | 659.10 | 7.37 |
| Stroop effect | 86.03 | 4.86 | 79.63 | 4.86 | 73.12 | 4.86 |
| Accuracy (%) |  |  |  |  |  |  |
| Neutral | 97.40 | 0.42 | 96.99 | 0.41 | 96.33 | 0.41 |
| Congruent | 97.20 | 0.36 | 97.94 | 0.36 | 96.81 | 0.36 |
| Incongruent | 92.72 | 0.68 | 92.68 | 0.68 | 90.72 | 0.67 |
| Stroop effect | −3.66 | 0.69 | −5.07 | 0.69 | −5.69 | 0.68 |
| P3 amplitudes (μV) |  |  |  |  |  |  |
| Neutral | 2.17 | 0.34 | 2.84 | 0.34 | 3.10 | 0.34 |
| Congruent | 2.46 | 0.34 | 4.61 | 0.35 | 3.66 | 0.34 |
| Incongruent | 2.63 | 0.31 | 3.64 | 0.32 | 3.22 | 0.31 |
| Stroop effect | −0.10 | 0.22 | −0.83 | 0.22 | −0.27 | 0.22 |
| HR (bpm) |  |  |  |  |  |  |
| Resting | 70.96 | 1.41 | 72.06 | 1.41 | 68.24 | 1.41 |
| Pre-treatment | 78.81 | 1.74 | 84.06 | 1.74 | 79.50 | 1.74 |
| Treatment | 122.12 | 1.43 | 131.84 | 1.43 | 77.67 | 1.43 |
| Post-treatment | 90.75 | 1.92 | 91.60 | 1.92 | 76.42 | 1.92 |
| RPE | 12.56 | 0.171 | 12.74 | 0.171 | - | - |
| Lactate (mmol·L^−1^) |  |  |  |  |  |  |
| Timepoint 1 | 2.56 | 0.20 | 2.50 | 0.20 | 2.27 | 0.20 |
| Timepoint 2 | 5.67 | 0.36 | 5.75 | 0.36 | 1.92 | 0.36 |
| Timepoint 3 | 9.02 | 0.46 | 4.75 | 0.46 | 2.66 | 0.46 |

CE, concurrent exercise group; AE, aerobic exercise group; CON, control group; HR, heart rate. RPE, rating of perceived exertion.
